# Nrf2 and Notch Signaling in Lung Cancer: Near the Crossroad

**DOI:** 10.1155/2016/7316492

**Published:** 2016-10-25

**Authors:** Angelo Sparaneo, Federico Pio Fabrizio, Lucia Anna Muscarella

**Affiliations:** Laboratory of Oncology, IRCCS “Casa Sollievo della Sofferenza” Hospital, San Giovanni Rotondo, Italy

## Abstract

The transcription factor Nrf2 (NF-E2 related factor 2) is a master regulator of the cell antioxidant response associated with tumor growth and resistance to cytotoxic treatments. In particular, Nrf2 induces upregulation of cytoprotective genes by interacting with the closely situated AREs (Antioxidant Response Elements) in response to endogenous or exogenous stress stimuli and takes part to several oncogenic signaling pathways. Among these, the crosstalk with Notch pathway has been shown to enhance cytoprotection and maintenance of cellular homeostasis, tissue organization by modulating cell proliferation kinetics, and stem cell self-renewal in several organs. The role of Notch and Nrf2 related pathways in tumorigenesis is highly variable and when they are both abnormally activated they can synergistically cause neoplastic proliferation by promoting cell survival, differentiation, invasion, and metastases.* NFE2L2*,* KEAP1*, and* NOTCH *genes family appear in the list of significantly mutated genes in tumors in both combined and individual sets, supporting the crucial role that the aberrant Nrf2-Notch crosstalk might have in cancerogenesis. In this review, we summarize current knowledge about the alterations of Nrf2 and Notch pathways and their reciprocal transcriptional regulation throughout tumorigenesis and progression of lung tumors, supporting the potentiality of putative biomarkers and therapeutic targets.

## 1. Introduction

Notch receptors (Notch1–Notch4) are a family of transmembrane proteins which interact with ligands of the Delta and/or Jagged/Serrate family. These receptors play a key role in the normal development of tissues and cell types, through diverse effects on differentiation, survival, and proliferation [1–3]. In tumors, Notch signaling has been observed to exert either oncogenic or antiproliferative effects within the mechanisms of cell invasion and metastases development.

The Nrf2 is a key regulator of the cell adaptive response to reactive oxygen species (ROS) and xenobiotics through the interaction with its master negative regulator, the Keap1 protein. Currently, the dark side of Nrf2 has emerged and growing evidences suggest that constitutive upregulation of Nrf2 is linked to cancer development and progression and contributes to chemo- and radioresistance.

Notch and Nrf2 are both transcription factors and their related pathways were discovered independently [4]. However, recent data have demonstrated the existence of a Nrf2-Notch crosstalk which supports cytoprotection and improves maintenance of cellular homeostasis and tissue organization. This review will mainly focus on the available scientific data which underlie the biological relevance of Nrf2 and Notch pathways and their crosstalk in lung tumors and suggest the potentiality of molecularly targeted agent combinations to overcome resistance to therapies.

## 2. Notch Signaling

*NOTCH* genes encode for highly conserved cell membrane receptors from* Drosophila* to humans that orchestrate a complex signaling pathway involving a number of ligands, negative and positive modifiers, and transcription factors [5]. In mammals, four Notch receptors (Notch1 to Notch4) and two families of Notch ligands (Jagged1 and Jagged2 and Delta-like-1, Delta-like-3, and Delta-like-4) have been described to play a critical role in the cell-contact-dependent cellular communications [2, 3, 6].

Although the overall structure of Notch receptors is similar, there are significant differences in the protein domains. The Notch1–4 receptors share an extracellular portion which contains a variable number of epidermal growth factor- (EGF-) like repeats: the Notch1 and Notch2 receptors contain 36 EGF repeats, whereas Notch3 contains 34 repeats and Notch4 contains 29 repeats. The other difference is in the transactivation domain (TAD). Notch1 and Notch2 contain a strong and a weak TAD, respectively, Notch3 has a potent but specific TAD best suited to the activation of the HES-5 promoter. In contrast, Notch4 does not contain a TAD. These structural differences may offer clues to the functional divergence among mammalian Notch paralogs [7].

The EGF-like repeats of extracellular portion of Notch are essential for ligand binding. The bond between ligands and extracellular Notch domains activates the intracellular portion and promotes intracellular sequential proteolytic cleavages by a metalloproteases of ADAM's family. Then the Notch intracellular domain (NICD) is released from the cytoplasmic membrane and translocates as active form into the nucleus, where it enhances the expression of several target genes encoding for Hairy Enhancer of Split (HES) family proteins, HES-related proteins (HEY), and p21cip1/waf1, cyclin D1 and 3, c-myc, and Her2, in a cell-context-dependent manner [3, 8, 9].

**Figure 1:**
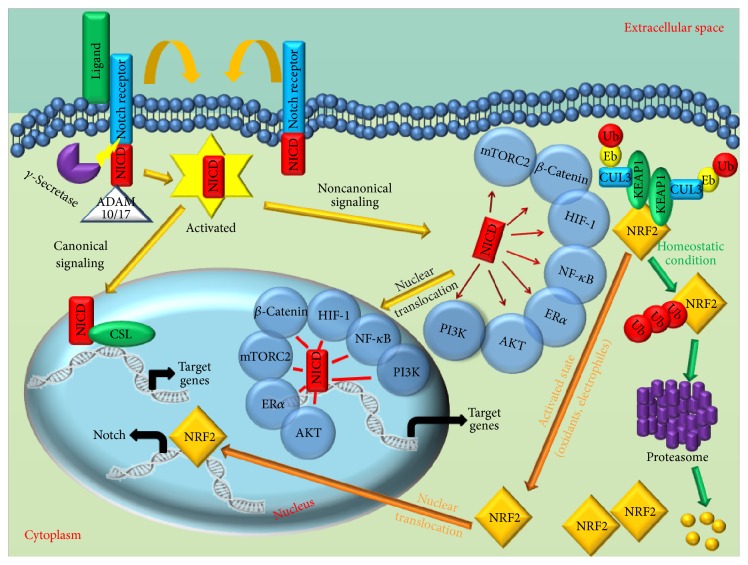
Canonical and noncanonical Notch signaling pathways. Notch signaling has a pleiotropic effect and is involved in cell survival, cell proliferation, cell metabolism, and differentiation. Canonical Notch pathway is primed by interaction of the Notch protein with a cell bound ligand. Upon interaction, Notch results cleaved, firstly by ADAM 10/17 protease and then by cleavage by the *γ*- secretase. Furthermore, Notch activated (NICD) translocates into nucleus and interacts with CSL protein, where, upon interaction, the proteins complex is converted into a transcriptional activator of targets genes. Noncanonical Notch pathways may be activated either dependently or independently of ligand interaction and may be *γ*-secretase dependent or independent. Noncanonical Notch signaling interacts with mTORC2, AKT, Wnt, HIF-1*α*, NF*κ*B, and PI3K pathways at either the cytoplasmic or nuclear levels. The gene regulatory region of the major Notch1 transcript has been described to possess a functional ARE through which Nrf2 can regulate Notch1 gene expression. In the activated state (orange arrow, transient upon stress stimuli or constitutive due to mutations in tumor cells),* de novo* synthesized Nrf2 protein accumulates into the nucleus, where it activates the transcription of several ARE-genes, including* NOTCH1*. In the basal state (green arrow), Keap1 binds Nrf2 and induces its ubiquitination. Upon ubiquitination, Nrf2 is degraded by proteasome complex.

Beside this canonical pathway activation, additional noncanonical Notch signaling pathways have been described. These additional pathways are independent from CSL (CBF1, Suppressor of Hairless, and Lag-1) transcription factor and related to other different transcription factors, such as beta-catenin, HIF-1a (hypoxia-inducible factor-1a), NF-kB (nuclear factor kappa-light-chain-enhancer of activated B cells), and estrogen receptor ER*α* (Figure 1) [10–13].

The Notch transcriptional machinery and signaling pathway are conserved among species, but in mammals this system shows the peculiarity to induce several, even opposite, biological effects depending on specific tissue types [4, 14]. Notch signaling networks can regulate a wide range of events in embryonic and postnatal development, including proliferation, apoptosis, border formation, and cell fate decisions. Aberrant expression of Notch receptors and Notch target genes have been reported in different human malignancies, including lung, skin, pancreas, breast, and colon cancers [15–20]. In lung tumors, depending on the subtype or specific molecular profiles, Notch family activity is often deregulated and activates several oncogenic pathways via direct or indirect induction [21, 22].

In a transgenic mouse model, Notch1 was overexpressed in the alveolar epithelium and induced alveolar hyperplasia and pulmonary adenomas through regulating type II lung epithelial cells. Moreover, the concomitant expression of MYC led to a progression to adenocarcinoma and metastases, indicating a synergistic effect between Notch1 and other oncogenes [23]. It has also been reported that Notch1 signaling plays a central role in the negative modulation of cell growth in lung adenocarcinoma through the ADAM metalloproteases and promotes apoptosis escape through a negative modulation of the p53 stability at protein level. These findings might explain the correlation between Notch1 activation and poor prognosis in NSCLC patients without* TP53* mutations [24–28]. Few data have been provided so far concerning the roles of Notch1 in lung adenocarcinoma harboring mutations in other lung cancer driver genes, such as* PIK3CA* or* EGFR* (Epidermal Growth Factor Receptor). In NSCLC cell lines, it has been preliminary observed that the expression of the active form of Notch1 (NICD1) leads to increased proliferation activity, malignant transformation, and tumor growth in presence of EGF (Epidermal Growth Factor), suggesting that EGFR activation may be essential for Notch-mediated malignant transformation and tumor growth [25].

Notch1 signaling has been shown to also act either as a negative or as a positive regulator of Phosphatase and Tensin Homologue gene (*PTEN*) transcription [29].* PTEN* downregulation is modulated by Notch1 through the activation of the transcription factor hair and enhancer of SPLIT (HES1), whereas* PTEN* upregulation derives from the inhibition of the binding protein suppressor of hairless (RBPJ), also known as CBF-1 [30–32]. In NSCLC and malignant mesothelioma cells, the activation of* PTEN* transcription by Notch1 upregulation has been observed to lead the prosurvival phosphatidylinositol 3-kinase (PI3K)/Akt/mammalian target of rapamycin (mTOR) signaling pathway [29–32].

In contrast with the role of Notch1 in promoting tumor initiation and progression, Notch2 shows a tumor suppressive activity by mediating cell differentiation in lung carcinogenesis. This evidence was also supported by immunohistochemical analysis of human NSCLC samples showing the loss or downregulation of Notch2 compared with normal lung tissues [33, 34]. In malignant mesothelioma (MM) cells Notch2 also appears to be diminished with a consequent decrease of toxic effects and a general benefit for cells [35]. Finally, the effect of Notch3 in lung carcinoma has been observed to be strongly dependent on cell type being a tumor suppressor in Small Cell Lung Cancer (SCLC) and a tumor promoting in NSCLC by differentially modulating cell adhesion, Epithelial Mesenchymal Transition (EMT), and cell motility [36].

Various studies have assessed the clinicopathological and prognostic value of Notch1 and Notch3 expression in NSCLC, but the results remain controversial. NSCLC tissues have significantly higher Notch1 protein levels compared to lung normal tissues, with strong variations in different studies and even within the same histotypes. Overall, Notch signaling can be suggested as a valuable biomarker to predict tumor progression in NSCLC. Overexpression of Notch1 and Notch3 has been associated with increased risk of lymph node metastasis and advanced TNM (tumor size, lymph nodes, and metastases) stages. Notch1 also represents an independent prognostic factor in surgically resected adenocarcinoma patients with a major impact in combination with VEGF-A (Vascular Epidermal Growth Factor-Alpha) upregulation [37, 38]. Future investigations might clarify the usefulness of targeting Notch signaling in specific subpopulation of NSCLC patients [39].

A key role of Notch signaling has been recently highlighted in the context of SCLC growth and resistance to therapy. Stable expression of the active form of Nocth1 in SCLC cells inhibits cell proliferation and decreases the expression of several neuroendocrine markers [40]. Moreover, alteration of Notch-Ascl1-Rb-p53 axis has been recently described as major driver of secondary transition from NSCLC to neuroendocrine phenotype and SCLC [41]. These findings provide a novel cellular mechanism for lung histology transition [42] and suggest Notch signaling reactivation as a possible therapeutic strategy for SCLC patients [43].

Finally, emerging evidences suggest that Notch signaling participates to the process of EMT, a highly coordinated process observed when epithelial cells lose some or most epithelial characteristics and acquire properties that are typical of mesenchymal cells. The transition of epithelial cells to mesenchymal cells is essential during embryogenesis and includes phenotypic changes such as loss of cell-cell adhesion, loss of cell polarity, and the acquisition of migratory and invasive properties. Accumulating evidences suggest that aberrant activation of the EMT developmental program contributes to tumor initiation, invasion, metastasis, and acquisition of therapeutic resistance [44, 45]. Notch, Wnt, Hedgehog (Hh), and TGF-b pathways induce well-differentiated epithelial cells to convert into motile mesenchymal cells in tumors via the activation of multiple EMT transcription factors, including Twist, Snail, Slug, and ZEB [46] and their deregulation correlates with poor clinical outcomes [47]. These findings corroborate the hypothesis that Notch1 and Notch3 may represent typical markers of stem-like cells indifferent solid tumors, including lung cancer [48].

## 3. Mechanisms of Notch Deregulation in Cancer

Notch receptors have been found deregulated in many tumors, and the prevalence and location of mutations within each Notch receptor coding gene varied considerably according to the tumor type [49]. Many identified mutations are heterozygous and correlate with a haploinsufficiency in tissue pattering and suggest that loss of a single copy functionally impairs signaling and therefore induces tumorigenesis. In general,* NOTCH1* gene mutations are more frequently recognized than in the other* NOTCH* receptor genes. This was in part, but not entirely, due to the greater number of tumors with Notch1 sequencing data. For head and neck cancer (HNSCC) and lung and breast cancers,* NOTCH1* mutations were relatively recurrent (5–15%) and clustered at or near identified important domains.

In lung cancer, the deregulation of the Notch pathway is mainly correlated with activating missense mutations mostly affecting the ligand-binding domain (EGF repeats 11 and 12) or the ankyrin domains which lead to a ligand-independent activation [50].* NOTCH1 *activating mutations have been defined as a common event in human NSCLC [51] and have been correlated to poor prognosis and response to therapy in lung patients without p53 mutations [52]. To note,* NOTCH1* mutations in SqCC appeared to be more frequent than pulmonary adenocarcinoma, and their typical location in close proximity of the ligand-binding domain leads to the speculation that Notch1 is more likely to function as a tumor suppressor in SqCC than in the adenocarcinoma histology [49]. However, the real frequency of* NOTCH1* mutations in NSCLC remains to be determined. The limited size and intrinsic variations of the just reported studied cohorts, along with the differences in sequencing strategy, do not allow a definitive conclusion on the magnitude of this event [53].

By contrast, mutations affecting the* NOTCH* family genes have been widely assessed and described as one of the most mutated pathways driving neuroendocrine features and SCLC. Different missense changes affecting all* NOTCH1–NOTCH4* genes with different frequency in relation to the different histologies of lung neuroendocrine tumors have been reported [54]. In SCLC, frequent damaging mutations have been identified in the extracellular domain with an incidence of about 25%, suggesting that Notch may act a tumor suppressor [55], leading to growth inhibition and neuroendocrine markers reduction [56].

Mutations in* NOTCH1–NOTCH4* family genes (28%) have also been recently reported in Large Cell Neuroendocrine Cancer (LCNEC) by genomic analysis. Many mutations were located in the extracellular EGF-like domain and were mainly associated with NSCLC-like subgroup but differ from the typical mutation pattern of lung adenocarcinoma. This represents an additional, strong evidence of the crucial role of Notch in lung neuroendocrine development [57].

In addition to direct mutations of* NOTCH* genes, alternative mechanisms of Notch deregulation have been reported in lung cancers. Molecular profiling of alternative splicing variants in lung adenocarcinoma have revealed frequent alternative splicing events affecting the* NUMB* gene, similar to primary breast and colon cancers. These abnormal isoforms lack normal activity and aberrantly induce the reduction of Numb protein expression levels and activation of the Notch signaling pathway there by promoting cell proliferation [58].

Finally, recent evidences indicate that there is a significant crosstalk between Notch and microRNAs. As a key component of the Notch-mediated transcription complex, Notch can regulate expression of a number of microRNAs; at the same time, Notch ligands, Notch receptors, or Notch effectors are regulated by microRNAs [59]. Indeed, members of five different families of miRNAs (miR-2, miR-4, miR-7, miR-11, and miR-79) have been shown to negatively regulate Notch target genes by recognizing conserved binding motifs within their transcripts [60]. However, few evidences about the role of this epigenetic mechanism of expression regulation have been provided in lung cancer. Pharmacological induction of miR-34a decreased the expression of Notch1 and its downstream targets including HES-1, Cyclin D1, Survivin, and Bcl-2, impairing Notch signaling, cell proliferation, and invasion and inducing apoptosis in NSCLC cells [61].

## 4. Nrf2 Signaling

**Figure 2:**
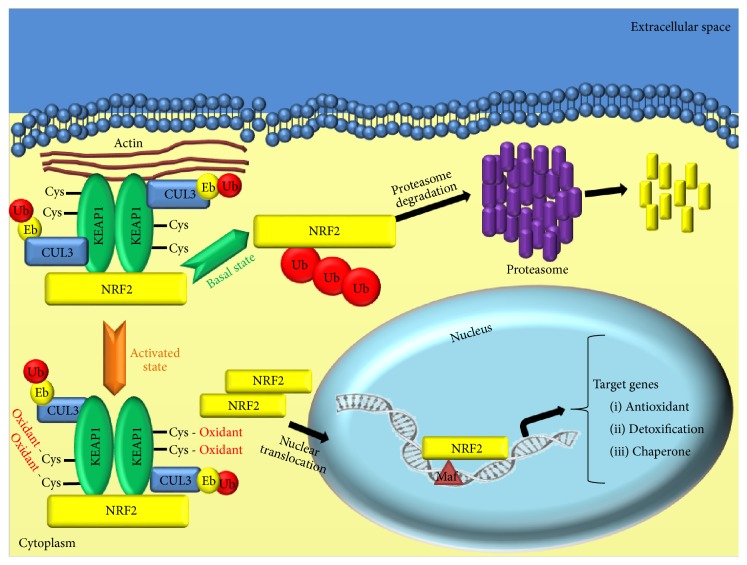
Keap1/Nrf2 axis in lung cancer. Under basal conditions (green arrow), Nrf2 is sequestered in the cytoplasm by the Keap1-Cul3 complex and rapidly degraded in the ubiquitin-proteasome dependent manner. This Keap1-mediated degradation activity requires two reactive cysteine residues of Keap1, located into the IVR domain. Upon stress stimuli (orange arrow), modification of these cysteine residues of Keap1 inhibits ubiquitin conjugation to Nrf2 by the Keap1-Cul3 complex, thereby provoking Nrf2-Keap1 impairment and resulting in the nuclear accumulation of* de novo* synthesized Nrf2 protein and enhancement of target genes transcription.

Nrf2 is a basic region-leucine zipper (bZIP) transcription factor that acts as a master modulator of cellular protection against carcinogens and oxidative damage in organisms. Although diverse mechanisms might be involved, it is speculated that the induction of phase II cytoprotective enzymes by Nrf2 chemical inducers occurs, at least in part, by modulating the activities of intracellular signaling kinases [62]. In the cellular basal state, the majority of* de novo* synthesized Nrf2 is repressed by physical interaction with Keap1, which is an adaptor protein to Cullin 3- (Cul3-) dependent ubiquitination and proteasomal degradation [63–67]. When cells are exposed to exogenous and endogenous toxic substances and to oxidative damage, a specific pattern of Keap1 cysteine modification arises [66]. By consequence, the Keap1 releases Nrf2 which translocates into the nucleus where it forms a heterodimer with small Maf proteins. This complex specifically recognizes enhancer sequences known as Antioxidant Response Elements (AREs), located in the regulatory regions of genes encoding for cellular defense enzymes, and activates their expression through the transcription machinery [68, 69]. Several Nrf2 target genes have been identified so far, and the number has increased through the recent technical advances [70]. Apart from the major cytoprotective functions of Nrf2 targeted genes, many of these genes also play in the context of oncogenesis, cell proliferation, apoptosis, and tumor cell growth in many cancer types (Figure 2). Recently, the involvement of Nrf2 has also been recognized in mitochondrial physiology as inductor of respiration substrates, membrane potential maintenance, integrity, and biogenesis [70–74].

Scientific findings in several neoplastic backgrounds underlined how the Nrf2 activity is clearly connected with oncogenic kinase pathways, structural proteins, hormonal regulation, other transcription factors, and epigenetic enzymes involved in the pathogenesis of tumors [75].

The large-scale genomic studies of NSCLC by The Cancer Genome Atlas (TCGA) consortium and others have supported that Nrf2 deregulation represents one of the major cancer driver pathways in the specific histotypes of SqCC where cigarette exposure can activate the oxidant stress response [76] and LCNEC of the lung with Non-Small-Cell Carcinoma features [77, 78]. Several mechanistic studies proved opposite roles of Nrf2 during carcinogenesis, either protective or promoting malignant progression [79]. The latter is supported by many clinical observations showing that constitutive upregulation is strongly associated with cancer development, progression, and resistance to conventional chemotherapy and radiotherapy in NSCLC [79–82]. Measuring nuclear Nrf2 abundance in NSCLC patients might be a useful index to predict the efficacy of platinum-based treatments. Nuclear accumulation of Nrf2 correlated with worse NSCLC cancer-specific survival and worse progress-free survival in three independent datasets of SqCC patients treated with surgery only [83–85]. As the main negative regulator of Nrf2, Keap1 activity and impairment also correlated with NSCLC survival. Our group discovered that NSCLC patients harboring* KEAP1* alterations had worse progression-free survival compared with other patients [86]. Similarly, Takahashi et al. found that* KEAP1* mutations caused an increase of Nrf2 expression in NSCLC patients and were correlated with worse progression-free and overall survival [87].

Along with* KEAP1* mutations, the expression levels of two Nrf2 downstream transcripts expressions, Ho-1 [88, 89] and Nqo1 [90–92], were found significantly associated with tumor invasiveness and patients survival in NSCLC advanced stage. In this regard, a recent extensive meta-analysis of microarray data for 240 Nrf2-mediated genes expression signature identify a group of 50 genes (NFE2L2-associated molecular signature, NAMS) that predicts a worse clinical outcome in 60% of NSCLC cohorts analyzed. These data corroborate the idea that NAMS could represent a promising prognostic biomarker in human lung cancer [93]. Correlation of Nrf2 downstream transcripts expression with tumor invasiveness and patients survival in NSCLC advanced stage has been also reported for Ho-1 [88, 89] and Nqo1 [90–92].

Three major crosstalks between Nrf2 and other classical oncogenic signaling pathways such as phosphatidylinositol 3-kinase (PI3K) [94], Kirsten retrovirus-associated DNA sequence (K-ras), [95] and Notch [4] have been reported in lung cancer as having a strong impact on tumor resistance outcome. The PI3K-Akt-mTOR pathway is commonly deregulated in several human malignancies including NSCLC [96], and activated PI3K signal increased accumulation of Nrf2 into the nucleus to enhance the transcription of enzymes involved in the pentose phosphate pathway [72]. Since radiotherapy agents can effectively induce apoptosis through generation of ROS [97] it was observed that specific PI3K inhibitor such as NVP-BKM120 can be used in SqCC to decrease Nrf2 protein levels and sensitize* NFE2L2* or* KEAP1*-mutant cells to radiation [94].* KRAS* gene mutations occur approximately in 20–30% of NSCLCs and confer to cancer cells resistance and survival [98, 99]. Promoter analysis showed that a TPA response element (TRE) located in exon1 of* NFE2L2* gene was activated by Kras. Thus, oncogenic Kras confers in NSCLC chemoresistance by upregulating Nrf2, enhancing the antitumor efficacy of cisplatin and providing a strong preclinical rationale to improve the management of lung tumors harboring* KRAS* mutations with Nrf2 pathway inhibitors [79, 95, 100].

## 5. Mechanisms of Nrf2 Deregulation in Cancer

Firstly described in NSCLC cell lines and tissues by Singh et al. in 2006, molecular impairment of Keap1/Nrf2 axis has been then extensively investigated in lung with different mutation clusters found to be related to specific histological subtypes. The overexpression of nuclear Nrf2 and the subsequent increase in the antioxidant defense in lung cancer cells are mainly related to genetic and epigenetic alterations of the* KEAP1* and* NFE2L2* genes [101]. Somatic mutations of the* KEAP1* gene frequently affect the DC domain and produce a decrease in Keap1-promoted Nrf2 ubiquitination by Cul3 or the impairment of nuclear export of Nrf2 by Keap1/Cul3 complexes. In both cases, under cellular stress condition, Nrf2 escapes degradation and translocates into the nucleus to induce the expression of its target genes [102–104]. Mutations in* NFE2L2* gene were also widely described in lung tumors, suggesting a strong link between molecular perturbations of the Nrf2 pathway and tissue exposure to ROS [105].* NFE2L2* mutations should determine a constitutive activation and have been found to mainly cluster within the DLG and ETGE motifs, which are hotspot sites for Nrf2 binding to the Keap1 DC binding domain. In particular, the ETGE mutant proteins are not ubiquitinated and concentrate in the nucleus, whereas mutations in the DLG resulting in the stabilization of Nrf2 increased its nuclear translocation and Nrf2* de novo* molecules synthesis [106, 107].

Mutations and copy number alterations of* NFE2L2* and* KEAP1* and/or deletion or mutation of* CUL3* were observed in 25–34% of SqCC among the classical alterations associated with this smoking-related histology subtype of lung cancer [77, 84]. Instead, a low incidence of* KEAP1 *mutations has been reported in advanced stage ADC patients with different ethnicity (3–19%) and a lower incidence of* EGFR* mutations [108, 109], except for papillary adenocarcinoma tumors subtypes (60%) [110]. In addition, TCGA analysis of lung adenocarcinomas has shown that the odds of a tumor carrying a* KEAP1* mutation increased more than sixfold among tumors with* LKB1* loss.* LKB1*-deficient tumors are susceptible to oxidative stress because they are unable to produce the appropriate adaptive responses in metabolism and biosynthesis. The high level of overlap in loss of function of* KEAP1 *and* LKB1* genes may suggest that selective pressure exists for the activation of Nrf2 as a secondary protective mechanism to compensate for* LKB1* loss [111].

More recently, new experimental evidences have demonstrated a mutual regulation between Nrf2 and microRNAs, especially in the mechanisms of tumor chemoresistance. Indeed, several miRNAs have been validated to target Nrf2 and thus affect its signaling pathway, although only few data have been collected in lung tumor [112–115]. On the other hand, Nrf2 has been demonstrated to regulate the expression of different miRNAs. For instance, functional studies in human lung fibroblasts reported as Nrf2/miR-140 signaling confers radioprotection by inducing Nrf2 nuclear translocation and subsequent activation of miR-140 transcription [116]. Moreover, miR-200a reactivation by histone deacetylation has been reported to destabilize Keap1 transcript in resistant lung tumor cell lines [117], whereas Nrf2-dependent regulation of miR-1 and miR-206 has been described to crucially promote non-small-cell lung proliferation and tumorigenesis by modulating the pentose phosphate pathway [118].

Lately,* KEAP1* alterations have emerged as an important molecular feature of neuroendocrine tumors of the lung. By performing genome/exome and transcriptome sequencing Fernandez-Cuesta et al. have demonstrated that it is possible to distinguish an LCNEC SCLC-like group, carrying* MYCL1* amplifications and mutations in both* RB1* and* TP53* genes from an AD/SQ-like group, harboring* CDKN2A* deletions,* TTF1* amplifications, and frequent mutations in* KEAP1* and* STK11*. This represents a picture of an evolutionary trunk that can branch to SCLC or AD/SQ on the basis of a different genetic background [112]. These data have been confirmed by Rekhtman et al., who reported an incidence of 31% of* KEAP1* mutations in LCNEC NSCLC-like subset [78].

In addition to somatic mutations, other mechanisms affecting Nrf2 expression in lung tumors have been found, even though this field still remains mostly unexplored. For instance, there are compelling evidences that epigenetic regulation might play a key role in modulating Keap1/Nrf2 axis in lung cancer cells [119]. Hypermethylation of the* KEAP1* promoter region was firstly described by Wang et al. as a pivotal mechanism in the modulation of the KEAP1 mRNA expression in cell lines and primary lung tumors that could be restored by 5-Aza treatment [120]. A larger study from our group on a cohort of resected primary NSCLCs confirmed these results and further proposed the epigenetic inactivation of* KEAP1* by promoter hypermethylation as the main mechanism which leads to reduced or absent Keap1 protein expression previously reported in NSCLC. Genetic and epigenetic analyses on this cohort suggest* KEAP1* biallelic inactivation as molecular marker of worst prognosis [86]. It has been recently demonstrated by* in vitro* analysis that the methylation status of* KEAP1* can also predict the tumor cells sensitivity to radiation. Importantly, when radiation is combined with the angiogenesis inhibitor Genestein, there is an increase of ROS levels and cell apoptosis via overexpression of Nrf2, GSS, and Ho-1 in lung adenocarcinoma cells [121]. A possible role of histone deacetylation/acetylation in the epigenetic regulation of the Keap1/Nrf2 pathway has been reported in human NSCLC, where hMOF-mediated acetylation of Nrf2 increased its nuclear retention and the transcription of its downstream genes, subsequently modulating tumor growth and drug resistance [122]. This new role of histone modification in the modulation of Nrf2 has been supported by Li et al., who showed that decreased Ezh2 expression significantly correlated with elevated expression of Nrf2 and its target genes, both in lung cancer tissues and in cell lines [113].

More recently, among the epigenetic mechanisms, new experimental evidences have demonstrated that miRNAs may crucially modulate the Nrf2 expression and affect its signaling pathway in a chemoresistance context [114–116, 119]. Nevertheless, most of the data have been reported in epithelial tumors such as breast and colon, whereas only few have been provided in lung cancer. On the other hand, functional studies on human lung fibroblasts reported as Nrf2/miR-140 signaling confer radioprotection by inducing Nrf2 nuclear translocation and subsequent activation of miR-140 transcription [117]. MiR-200a reactivation by histone deacetylation has been reported to destabilize Keap1 transcript in resistant lung tumor cell lines [118], whereas Nrf2-dependent regulation of miR-1 and miR-206 has described as crucial in non-small-cell lung proliferation and tumorigenesis through the modulation of the pentose phosphate pathway [123].

## 6. Nrf2-Notch Pathways Crosstalk in Lung Cancer

Nrf2, Keap1, and Notch1 rank among the first frequently mutated genes in tumors and were deemed to be significant both in the combined sets of tumors and in individual tumor types. This observation leads to the speculative general notion that the outcome from aberrant Nrf2-Notch crosstalk by molecular impairment in these genes might enhance tumorigenesis and progression to cancer [4], especially in the stem cell (SC) context.

A number of experimental models have been employed to demonstrate that Nrf2 is involved in the maintenance of the stem cell phenotype. ROS have more recently been found to have useful roles in SC proliferation and differentiation [129]. However, the functional significance of the ROS status in different types of SCs, the downstream signaling events, and the role of ROS in SC self-renewal for repair and homeostasis is controversial [130–132]. In* Drosophila* intestinal stem cells, loss of the CncCbZIP-CNC (cap-n-collar subfamily of basic leucine zipper) transcription factor has been reported to increase ROS levels and cell proliferation rates, suggesting that CncC is required to keep the intestinal stem cells in a state of quiescence and to prevent them from entering the cell cycle [130]. In mouse hematopoietic stem cells, loss of Nrf2 has been shown to lead to an expansion of the progenitor pool of myeloid and lymphoid lineages, again suggesting that Nrf2 supports stem cell renewal and proliferative quiescence [133]. A reciprocal Nrf2-Notch transcriptional regulation has been described in hepatobiliary system, having a key role in liver development and in maintenance of hepatic function and its deregulation might be one of the main pathways for promoting cancer [134–136].

Less evidences have been provided for a clear Notch-Nrf2 crosstalk in lung cancer. The airway epithelium is constantly exposed to environmental oxidants and therefore serves as an interesting model system to study redox signaling. Cigarette smoke is known to cause oxidative stress-induced airway injury [137], diseases, and cancer airway-related through well-known mechanisms [138].

**Figure 3:**
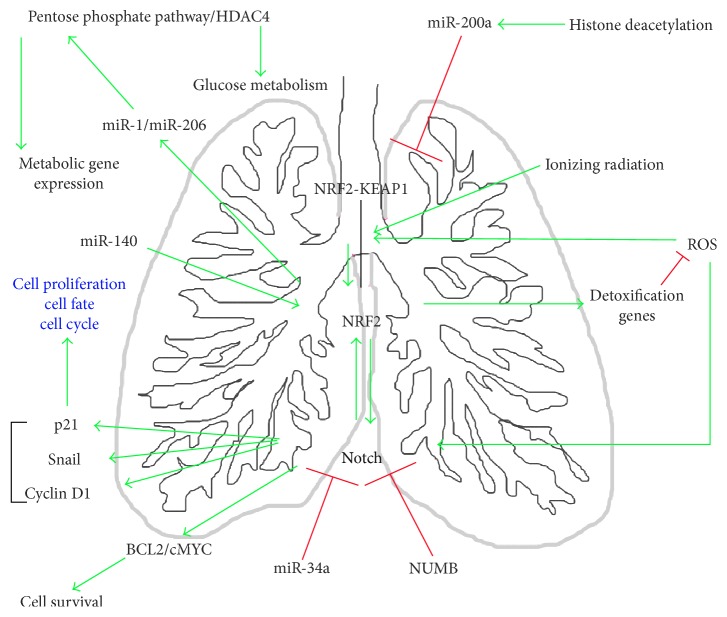
Integrated network of Nrf2-Notch crosstalk in lung. Links for biological events occurring in lung involving Nrf2 and Notch interaction with other signaling networks. Black lines indicate interaction, green arrows indicate activation, and red arrows indicate repression.

Additionally, Nrf2 has been implicated in the self-renewal of human airway basal stem cells, but in this case the flux of ROS levels appeared to be the critical factor. In the same context, Notch1 signaling pathway was implicated in helping dynamic changes in ROS levels [138] and has been noted to be essential in early lung development and in the regulation of stem cell self-renewal; thus, when abnormally activated these pathways can cause neoplastic proliferation, representing an early event in tumorigenesis [139]. Beside this, an inverse modulation of Notch by Nrf2 was observed. The gene regulatory region of the major Notch1 transcript has been described to possess a functional ARE region through which Nrf2 can directly regulate Notch1 gene expression, thus promoting airway basal stem cells' self-renewal [138, 140]. Finally, recent data have shown that Nrf2 strongly regulates Notch1 activity and promotes radiation-induced apoptosis through Nrf2 mediated Notch1 signaling in NSCLC cells. Thus, Notch signaling is an important determinant in radioresistance of lung cancer cells [141] (Figure 3).

An indirect suggestion of a functional interdependence in the Notch and Nrf2 pathways comes from recently published studies of genomic analysis in LCNECs. Genes mutation profiling revealed a high incidence of* NOTCH* genes family (33%) and* KEAP1-NFE2L2* (39%) alterations in specific subsets of LCNECs. In particular,* NOTCH* genes family alterations represent one of the most relevant differences in NSCLC-like LCNEC from classic lung adenocarcinoma and are of particular interest because they give a strong evidence for their crucial role of Notch receptors in neuroendocrine fate specifications in normal and tumor development [55, 57]. However, despite the overall similarity, the most relevant differences identified in SCLC-like LCNEC were an elevated rate of* KEAP1-NFE2L2* mutations that rarely occur in conventional SCLC but are frequent in SqCC, suggesting a stronger histogenetic relationship of some conventional SCLCs and SqCCs [78]. Finally, frequent cooccurring mutations* NFE2L2*,* KEAP1*, and* NOTCH1* in a study on more than four thousand human cancers support the notion that the outcome from aberrant Nrf2–Notch crosstalk by mutations in these genes might specifically enhance tumorigenesis and progression to cancer [142].

## 7. Therapeutic Targeting of Notch and Nrf2 Pathways

The central role of Notch signaling in cancer, cancer stem cell maintenance, and angiogenesis has significantly fostered the transition from preclinical research into clinical application of alternative targeted compounds, including small molecule inhibitors and large mAbs (monoclonal antibodies) targeting Notch signaling [143]. At the same time, the adjuvant effect of these inhibitors in combination with current chemotherapeutics is still under evaluation in different clinical trials (Table 1) [144].

The interaction between Notch receptor(s) and ligand(s) takes place within a tight cell-to-cell compartment. Following ligand-receptor association, the sequential two-steps cleavage by the ADAM/TACE proteinase and *γ*-secretase, respectively, culminates in the functional activation of Notch signaling. Numerous preclinical models have documented so far the anticancer effects of different classes of compounds inhibiting Notch signaling activation such as siRNAs, GSIs, and mAbs [145], with encouraging results for clinical implementation in combination with either chemotherapy or targeted agents [146]. Among these, the oral GSI PF-0308414 showed clinical activity in a phase I study in patients with advanced stage solid tumors [147].

In this context, Notch pathway inhibition is currently under investigation as novel therapeutic option of SCLC. For instance, the fully human IgG2 antibody Tarextumab (TRXT, OMP59R5) combined with chemotherapy has been shown to significantly reduce tumor recurrence in patient-derived SCLC xenografts, by targeting Notch2/Notch3 [148]. On the strength of these results, a phase I/II study of Tarextumab in combination with six cycles of cisplatin and etoposide in ES-SCLC, followed by Tarextumab maintenance (PINNACLE, NCT01859741), is currently ongoing [149].

Alternative approaches for targeting Notch signaling in lung cancer may include several natural agents, such as curcumin (3,3′-diindolylmethane, DIM), resveratrol 3,5-bis (2,4-difluorobenzylidene)-4-piperidone (DiFiD), and epigallocatechin-3-gallate (EGCG) [150], whose anticancer activity has been demonstrated in both* in vitro* and* in vivo* models of other solid tumors [151, 152].

Similarly, the pharmacological inhibition of Nrf2 signaling may represent a further therapeutic strategy for cancer treatment, especially in those patients carrying increased levels of Nrf2 (Table 2). Indeed, recent reports have demonstrated that the high levels of Nrf2 are significantly associated to chemo- and radioresistance, rendering the development of novel Nrf2 inhibitors particularly intriguing [153]. For example, it has been demonstrated that all-trans retinoic acid (ATRA) and retinoic acid receptor-*α* (RAR*α*) agonists can directly sequester Nrf2 and prevent its binding to the ARE, leading to the global downregulation of Nrf2-dependent gene expression [154]. Similar outcomes have been reported as a result of the physical blocking of Nrf2 operated by other nuclear receptors, such as peroxisome proliferator-activated receptor-*γ* (PPAR*γ*), estrogen receptor-*α* (ER*α*), estrogen-related receptor-*β* (ERR*β*), and glucocorticoid receptor (GR) [155].

An increasing number of natural compounds are known to also exert a strong effect on Nrf2, thus corroborating the idea of a cross-link with Notch pathway. Among these, sulforaphane thereby induces an activation of the Nrf2/Keap1 cellular detoxification cascade by reacting with thiols of Keap1 DGR domain [156, 157], whereas benzo(a)pyrene(B(a)P) has been shown to inhibits carcinogenesis process in lung mouse model by promoting ROS-mediated apoptosis [158, 159]. Similar mechanism of action has been observed with Oltiplraz, known as a dithiolthione substitute able to induce phase II enzymes, which exhibited a chemoprevention effect in mouse lung adenocarcinoma [160, 161]. Resveratrol restored cigarette smoke exposure- (CSE-) depleted GSS (glutathione synthetase) levels by upregulating GCL (*γ*-glutamate cysteine ligase) by reducing CSE-mediated Nrf2 modifications [162]. Intriguingly, many studies has shown that curcumin, a natural phenolic compound, which is extracted from a member of the ginger family, has a dually role as inhibitor of Notch1 in osteosarcoma cells and a Nrf2 activator in normal tissues [163, 164].

In conclusion, the therapeutic Nrf2 targeting holds great promise for the treatment of lung cancers especially because it has documented a beneficial adjuvant effect in combination with any category of chemotherapeutics, both ROS generating and non-ROS generating agents. To date, one limitation is represented by the lack of few selective inhibitors for Nrf2 and related pathway. Another limitation is represented by the high risk of off-target toxic effects that most of the Nrf2-targeting drugs may generate due to unspecific interactions with other proteins by their electrophilic surface [165]. Nevertheless, the rational design of nonreactive small molecules directly targeting the Keap1-Nrf2 pathway appears to be the most promising strategy to limit the toxic effects often related to indirect inhibitors and increases stability and bioavailability, as compared with peptide inhibitors [166, 167].

## 8. Concluding Remarks

There are compelling evidences that developmental pathways, including Notch, act in concert with other pathways such as Nrf2 pathway involved in resistance to therapy, rather than as a simple on-off switch. Notch can play as tumor suppressor or oncogene depending on the cell type [168] and interestingly, Nrf2 likely functions in a similar fashion [169]. Nrf2 acts as a prosurvival factor through the expression of its cytoprotective target genes, and molecular deregulation of either Nrf2 or Keap1 is widely described in lung cancer, such as Notch family impairment. In tumors, Nrf2 and Notch signaling pathways appear to mutually regulate each other in which Notch1 is an Nrf2 target gene and Nrf2 is a RBPjk target gene. The roles of the Nrf2-Notch bidirectional interaction in driving or impeding a tumor lung phenotype are still unclear mainly in the context of stem cell renewal and cell proliferation and differentiation. Pharmacological interventions based on these transcription factors collaboration are demanded close with a further explorations of the regulation of this crosstalk in cellular and lung tissue context.

## Figures and Tables

**Table 1:** Details of selected trials with therapeutic Notch-targeting agents for lung cancer treatment.

Class	Mechanism	Drug	Target	Condition	Development phase	Trial status/ID
mAbs to Notch receptors or ligands (neutralizing antibody)	Interference with ligand-induced Notch subunit separation and Notch ligands	OMP21M18 + CP^*∗*^	DLL4 (Delta-like ligand 4)	Nonsquamous NSCLC	Ib	Active, not recruiting/NCT01189968
		OMP-59R5 + EP^*∗*^/CDDP^*∗*^ or CBDCA^*∗*^	Notch2/Notch3	Stage IV Small Cell Lung Cancer (SCLC)	Ib/II	Recruiting/NCT01859741

mAbs to Wnt signaling pathway (monoclonal antibody)	Blocking canonical WNT/*β*-catenin signaling through binding of five FZD receptor	OMP-18R5 + DTX^*∗*^	Wnt cancer stem cell pathway	Recurrent or advanced NSCLC	Ib	Recruiting/NCT01957007

*γ*-Secretase inhibitor (GSI)	Inhibition of final Notch cleavage by *γ*-secretase	RO4929097	Notch1, Notch2, Notch3, Notch4, Notch ligands	Recurrent NSCLC Stage IIIB NSCLC Stage IV NSCLC	II	Terminated/NCT01193868
		RO4929097 + OSI-774^*∗*^		Recurrent NSCLC Stage IV NSCLC	I	Terminated/NCT01193881
		BMS-906024		Advanced/metastatic squamous NSCLC	I	Ongoing/NCT01292655
		BMS-906024 + chemotherapy regimens^*∗*^		Advanced/metastatic squamous NSCLC	Ib	Ongoing/NCT01653470
		BMS-906024 + PTX^*∗*^		NSCLC	I	Synergistic antitumor activity of the Notch gamma secretase inhibitor BMS-906024 and paclitaxel in the treatment of lung adenocarcinoma. Abstract 2535, AA

*γ*-Secretase inhibitor (GSI)/VEGFR tyrosine kinase inhibitor	Inhibit of final Notch cleavage by *γ*-secretase and stop the growth of tumor cells by blocking some of the enzymes needed for cell growth	RO4929097 + AZD2171		Recurrent NSCLC Stage IIIA NSCLC Stage IIIB NSCLC Stage IV NSCLC	I	Completed/NCT01131234

^*∗*^Chemotherapy agent: CP (carboplatin plus paclitaxel); EP (etoposide), CDDP (cisplatin), CBDCA (carboplatin); DTX (docetaxel); OSI-774 (Erlotinib); chemoregimens: PTX (paclitaxel), 5-FU plus irinotecan (FOLFIRI), or CP (carboplatin plus paclitaxel).

Each status of development phases results from https://www.clinicaltrials.gov/.

**Table 2:** Details of selected trials or scientific reports on therapeutic Nrf2-inhibition for lung cancer treatment.

Class	Mechanism	Drug	Target	Condition	Development phase	Trial status/ID or scientific reports
Vitamin A metabolite	All-trans retinoic inhibits the basal and inducible activity of Nrf2	13-CRA + IFN-A^+^	Retinoid X receptor alpha binding to Neh7 domain of Nrf2	Recurrent Squamous Cell Lung Cancer (SqCC)	II	Completed/NCT00002506
	RAR-alpha complex (with Nrf2) is not able to bind to ARE and decreases the Nrf2 ability to activate ARE-driven genes	ATRA + CDDP^*∗*^ + MTC^*∗*^ + NVB^*∗*^		Stage IIIB or IV NSCLC	II	Unknown/NCT00005825
		13-CRA + IFN-A^+^ + PTX^*∗*^		Recurrent Small Cell Lung Cancer (SCLC)	II	Completed/NCT00062010
		ATRA + PCB^*∗∗∗*^		Stage IIIB or IV NSCLC^*∗∗*^	II	Completed/NCT01048645

Quinoid diterpene	Inducing apoptosis by sensitizing A549/DDP cell and inhibiting Nrf2 pathway in chemoresistant lung carcinoma	CTS + CDDP^*∗*^	Inhibitor of STAT3 and AChE	A549/DDP cell line	*In vitro* and *in vivo*	Xia et al., 2015 [124], Cell PhysiolBiochem
Flavonoid	Inhibiting ARE-driven gene expression redox-independently, leading to a dramatic decrease in Nrf2 protein levels with depletion of reduced glutathione	LUT	SRC tyrosine kinase	A549 adenocarcinoma cell line	*In vitro* and *in vivo*	Tang et al., 2011 [125], Free Radical Biology & Medicine
	Cell proliferation, the expression of Nrf2, and antioxidant enzyme were all reduced in tumor xenograft tissues after cotreatment and inhibiting tumor cell growth	LUT + CDDP^*∗*^		A549 cell line in athymic nude mice	*In vitro* and *in vivo*	Chian et al., 2014 [126], Biochemical and Biophysical Research Communication

Glycopeptide antibiotic	Involveing suppression of Nrf2 activation, inhibiting the incorporation of thymidine into DNA strand, and causing cell cycle arrest in G2 and in mitosis	BLM + CDDP^*∗*^ + 5-FU^*∗*^ +	Synthesis of nucleic acid	A549 adenocarcinoma cell line LC-AI squamous cell line NCI-H292 mucoepidermoid cell line	*In vitro*	Homma et al., 2009 [127], Clin Cancer Res

Quassinoids	Inhibiting the Nrf2-mediated protective response at subnanomolar concentration, increase ubiquitination, enhancing Nrf2 degradation, and reducing Nrf2 protein levels	Brusatol	Formation of the first peptide bond between puromycin and methionyl-transfer RNA	A549 cell line	*In vitro* and *in vivo*	Vartanian et al., 2016 [128], Molecular & Cellular Proteomics
	Cotreatment inhibits the Nrf2 protective mechanism, leads to decreases cell proliferation, enhances oxidative DNA damage, and reduces apoptosis	Brusatol + CDDP^*∗*^		Cell culture and murine A549 xenograft models	*In vitro* and *in vivo*	Tao et al. [95], Cancer Res

AA panel of Nrf2 inhibitor cited in the table as follows: 13-CRA (13-*cis*-retinoic acid), ATRA (all-trans retinoic acid); CTS (cryptotanshinone); LUT (luteolin); BLM (bleomycin); brusatol.

^+^Biological agent: IFN-A (interferon alpha).

^*∗*^Chemotherapy agent: CDDP (cisplatin), MTC (mitomycin C), NVB (vinorelbine tartrate); PTX (paclitaxel); 5-FU (fluorouracil).

^*∗∗*^ In this study patients that have already received paclitaxel and cisplatin (PC) were recruited.

^*∗∗∗*^PCB (placebo) means an innocuous medication given to the control group in experiments on the efficacy of a drug.

Each status of development phases results from https://www.clinicaltrials.gov/.
